# Developing a Dynamic Supervision Mechanism to Improve Construction Safety Investment Supervision Efficiency in China: Theoretical Simulation of Evolutionary Game Process

**DOI:** 10.3390/ijerph18073594

**Published:** 2021-03-30

**Authors:** Shitao Gong, Xin Gao, Zhou Li, Linyan Chen

**Affiliations:** 1 School of Economics and Management, Tongji University, Shanghai 200092, China; 1810250@tongji.edu.cn (S.G.); gaoxin@tongji.edu.cn (X.G.); linyan.chen@connect.polyu.hk (L.C.); 2 Department of Building and Real Estate, The Hong Kong Polytechnic University, Hong Kong 999077, China

**Keywords:** health and safety, supervision, safety investment, evolutionary game, construction industry

## Abstract

The construction industry suffers from poor safety performance caused by the joint effect of insufficient safety investment by contractors and inefficient safety supervision by the government because of the information gap between the two sides. The present study aims to put forward a new pathway to improve safety investment supervision efficiency and analyze the decision-making interactions of stakeholders under this new pathway. For this purpose, this study establishes a safety investment information system to eliminate the information gap between the government and contractors for construction projects in China and further develops a dynamic safety investment supervision mechanism based on this. Evolutionary game theory is used to describe the decision-making interactions among stakeholders under the current static supervision mechanism and the dynamic supervision mechanism proposed in this research. Moreover, system dynamics is adopted to simulate the evolutionary game process and analyze the supervision effect and equilibrium state of different supervision mechanisms. The results reveal that the proposed safety investment information system could facilitate the transition of the supervision mode from static to dynamic; the evolutionarily stable strategy does not exist in the current static penalty scenario; and the dynamic supervision mechanism that correlates penalties with contractors’ unlawful behavior probability can restrain the fluctuation of the evolutionary game model effectively and the players’ strategy choices gradually stabilize in the equilibrium state. The results validate the effectiveness of the proposed dynamic supervision mechanism in improving supervision efficiency. This study not only contributes to the literature on safety supervision policy-making but also helps to improve supervision efficiency in practice.

## 1. Introduction

The construction industry is deemed as one of the most dangerous industries due to its complicated construction environment, frequent use of heavy equipment and inevitable hazardous worker interactions [1]. According to prior studies, 22% of occupational fatalities in America, 27.2% in Britain, and 27.6% in Korea occur in the construction industry [2,3,4]. In China, a total of 3843 fatal injuries were occurred at construction sites in 2017, which accounts for 34.3% of all workplace fatalities [5]. These high casualty rates reflect the poor safety performance in the construction industry around the world [6,7], which has become a significant public health problem. Therefore, it is essential to improve occupational health and safety as well as safety performance in the industry [8]. Many studies have been performed to investigate the key factors in determining construction safety performance, and current research has reached a consensus that the safety performance of construction projects is the result of the interactions between the contractor’s internal safety investment and the government’s external safety supervision [9,10,11].

Previous studies confirmed the positive effect of safety investment on safety performance [12,13] and showed that an increase in safety investment leads to better safety performance at construction sites. However, because safety investment is usually a part of the project contract sum in the construction industry and the specific structure of the industry is characterized by high competition, low profit margins and competitive tendering, construction contractors may unavoidably conduct a cost-safety trade-off in the bidding and construction stages, which leads to a common situation of insufficient safety investment in the construction process [14]. Under these circumstances, government supervision has been considered a key constraint for safety decision-making by contractors [15] and plays a significant role in promoting safety performance in the construction industry [11,15,16,17]. Efficient government supervision requires a high level of information transparency related to safety production between the government and contractors [18]. Based on adequate and real-time safety production information, the government can adjust its supervision strategies dynamically in response to varying degrees of illegal contractor behaviors and then realize the rapid response to the problems encountered in the construction process [11,19]. Nevertheless, the safety environment of construction sites is dynamic and complex and presents variations in time, cost and quality as well as interactions among different construction stakeholders [20]. Delays and deficiencies in communication occur between the government and contractors, resulting in an information gap that weakens supervision efficiency [21,22].

Therefore, it is important to address the issue of inefficient supervision caused by the information gap between stakeholders. However, studies on this issue are far from complete. Although some studies have explored optimal safety supervision decision-making [23,24], few studies have been conducted from a dynamic perspective in which the government adjusts its supervision strategies dynamically according to the real-time safety production status based on a high level of information transparency between stakeholders. Meanwhile, although some studies have proposed corresponding information systems to strengthen the information communication between the stakeholders [25,26], but these studies have usually paid attention to the technical aspects of developing an information system, and how the information system influences the stakeholders’ decision-making in the long-term safety supervision process remains largely unknown. Moreover, although evolutionary game theory has been widely applied to model the decision-making interactions between stakeholders in the construction safety supervision process, previous studies have usually concentrated on analyzing the game process between two stakeholders [26,27] and few have focused on the evolutionary game analysis of multiplayer scenarios.

This study aims to answer two questions. The first question is how to eliminate the information gap between the government and contractors in the safety investment supervision process, and explore a new pathway to improve supervision efficiency. The second question is to analyze the decision-making interactions of stakeholders under this new pathway and ascertain the stakeholders’ behavior characteristics. To bridge the research gap and answer the questions, this study establishes a safety investment information system aimed at filling the information gap between government and contractors and further develops a dynamic safety investment supervision mechanism based on this. The decision-making interactions between stakeholders under the dynamic supervision mechanism is modeled by multiplayer evolutionary game theory, and the game process is simulated by system dynamics (SD). The main innovation points of this study are as follows: (1) different from the existing research that normally neglects the dynamics of supervision process, we consider the government supervision in a dynamic perspective in which the government’s supervision strategies are flexible in response to the safety production status on sites; (2) not only establishing an information system, but how the information system influences the decision-making of stakeholders are also explored; and (3) interactions between the government and contractors as well as interactions between multiple contractors are considered in the proposed model, and the combination of evolutionary game theory with SD simulation addresses the issue of multiplayer interactions, which has been rarely addressed in previous studies. According to the results, this study has implications for governments in optimizing safety supervision decision-making to improve supervision efficiency. It also helps to restrict the behavior of insufficient safety investment by contractors, which will lead to a safer working environment on construction sites.

This paper is organized as follows: Section 2 reviews the previous studies about safety investment and safety supervision; Section 3 illustrates the research methodology of this study; Section 4 presents the research results; Section 5 introduces further analyses and discusses the results; and Section 6 draws several main conclusions.

## 2. Literature Review

### 2.1. Safety Investment in the Construction Industry

The concept of safety investment is often confused with safety cost in the construction safety research domain. According to the definition proposed by Lopez-Alonso et al., the construction safety costs involve the cost of the services, goods and resources used to decrease the frequency of construction accidents, as well as the cost result from the occurrence of accidents [28]. Safety costs can be divided into two categories: accident prevention costs and accident occurrence costs [29]. Accident prevention costs are those spent by contractors to carry out health and safety measures on construction sites. Currently, the accident prevention cost is also called safety investment [15,29]. Therefore, safety investment is included in the scope of safety costs and constitutes part of these costs. Safety investments (accident prevention costs) can be divided into mandatory and voluntary part. Mandatory investments are costs used to implement the minimum health and safety measures that are required by laws and regulations, which involves safety staffing, safety training, necessary equipment and facilities [30]. Voluntary investments are those not compulsorily required by laws and regulations, and they are often driven by the contractor itself and generally contain safety committees, safety promotions and incentives as well as new technologies, methods or tools developed for safety [31].

Some existing studies have proposed a popular assumption that increasing safety investment can generate a better safety performance [13,32], which can be indicated by various indicators, such as the casualty rate and accident rate [6,7]. After investigating 40 construction projects in progress, López-Alonso et al. revealed that the number of accidents in the projects is negatively correlated with the level of safety investment [12]. Based on Monte Carlo simulation, Shohet et al. found that increasing the safety investment of construction projects by 0.5%, from 0.5% to 1.0%, would result in a marginal benefit of 164,700 USD [33]. These findings verify the popular assumption. Furthermore, studies conducted from a bottom-up perspective to investigate how different safety investment categories could affect construction safety performance provided more detailed evidence. Han et al. proposed that increases in tangible safety investment (e.g., incentive) generated stronger motivations for employees to enhance their behavioral safety performance [34]. Oswald et al. suggested that the construction industry should invest more in safety and forward-thinking companies should exceed the minimum standards required by safety regulations and invest in voluntary accident prevention costs [14].

Safety investment usually has significant effects on the overall budget of a project [14]. Therefore, the optimal safety investment level ought to strike a balance between reducing the accident rate and controlling total costs [15]. By conducting a cost-benefit analysis, Hallowell indicated the existence of a critical point where extra investment produces diminishing returns [35]. The study from opportunity cost perspective conducted by Ma et al. revealed that the optimal safety investment level is determined by minimizing expected total opportunity costs, which consists of shortage costs and excess costs. The former resulted from the occurrence of accidents and the latter resulted from investing more than required for accident prevention [15]. However, in practice, the construction industry is a heavily oversupplied market and uses competitive tendering as the principal procurement approach [36]. Regarding this procurement approach, clients primarily select their preferred contractors based on a ‘lowest price policy’ in which the lowest bidder wins the contract [37]. Thus, to stay economically competitive and reach the maximum profits, many contractors have few motivations to invest adequately in construction safety [14,38]. A field survey conducted by Shohet et al. on 30 construction projects revealed that the preventive safety activity resources actually invested by contractors are on average only half of the optimal amount [33]. The issue of insufficient safety investment may undermine the safety performance of practical construction projects.

### 2.2. Government Safety Supervision in the Construction Industry

Government and contractor safety efforts are strategic complements to some extent [9,10]. Government safety supervision in the construction industry involves two aspects: establishing a compulsory safety standard for companies to make sure every company meets a minimum safety requirement [39]; and regularly inspecting and evaluating the safety conditions of the construction sites in every company [15]. Heinrich’s accident causation theory implied that management is responsible for preventing accidents [40]. Recent research has also widely validated the pivotal role of government supervision in improving workplace safety. Based on the analysis of 42 accident cases, Lu and Zhang found that unfavorable government supervision is one of the critical safety risk factors during the metro construction phase [41]. Besides, in coal mine industry, Chen et al. revealed that the establishment of safety supervision institutions could noticeably reduce the industry’s death rate [42].

Studies have been conducted to improve safety supervision decision-making for the government. Zhu and Li pointed out that punishment is an impactful method to restrict contractors’ illegal behaviors and guarantee construction safety [43]. Similarly, Ma and Zhao found that the increase of unit penalty cost can decrease the probability of workplace accidents [23]. Wang et al. indicated that the establishment of a safety supervision system should consider both internal and external safety environments and that the most predominant strategies for establishing the safety supervision system lie in safety culture, organizational structure and safety performance evaluation [24]. In terms of research methods, Hausken and Zhuang adopted game theory to analyze the interactions between the enterprise’s safety effort and government’s safety supervision [9,10]. Similar studies have been performed by Pi et al. [26] and Cheng and Chen [27], and they mainly focused on game analysis between two stakeholders.

However, in reality, the effectiveness of supervision is reduced because of the information gap between the government and contractors [21,22]. Zhang et al. indicated that the information gap can hinder the information exchange and receiving supervision feedback between the government and contractors in the safety supervision process [44]. To address this issue, a few scholars have developed corresponding information systems to strengthen the information communication between the government and construction contractors during the safety supervision process. Park et al. proposed a web-based construction safety management information system to improve safety management at construction sites [25]. Pi et al. proposed a safety management blacklist system, which serves as an effective supplement to government supervision [26]. Fargnoli and Lombardi summarized that the establishment of safety supervision information systems based on emerging technologies gradually becomes a promising direction for further construction safety supervision, which not only helps to eliminate the information gap between government and contractors but also achieves a rapid-response operation mechanism for the problems encountered in the supervision process [45]. 

In summary, there are some limitations in previous research. First, existing research normally considers the government’s safety supervision decision-making is static and neglects the dynamics of supervision process. Second, studies on developing safety supervision information system usually concentrated on the technical aspects, and few to explore the influence of information system on stakeholders’ decision-making. Finally, previous studies mainly focused on the decision-making interactions between the government and contractors in the supervision process, and the interactions between multiple contractors were less explored. Therefore, this study is going to put forward a more efficient supervision mechanism based on emerging information technologies and further explore the decision-making behavior characteristics of multiple stakeholders under this new supervision mechanism. 

## 3. Research Methodology

### 3.1. Overall Research Framework

**Figure 1:**
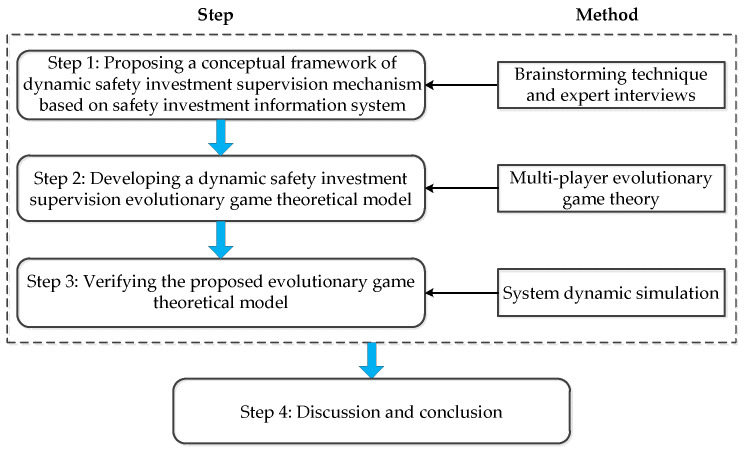
Flow of the overall research framework.

As we have mentioned in the introduction, this study aims to establish a dynamic safety investment supervision mechanism to optimize the government’s supervision decision-making. Therefore, a dynamic safety investment supervision mechanism based on a safety investment information system was proposed in the first step. Afterwards, a multiplayer evolutionary game theoretical model was further developed to describe the complex long-term decision-making process of stakeholders under the dynamic supervision mechanism. In the third step, the evolutionary game model was simulated by SD to analyze and compare the supervision effect and game equilibrium state of different supervision strategies. Moreover, further discussion on the simulation results and several main conclusions were drawn based on the above analysis. Figure 1 shows the flow of the overall research framework.

### 3.2. Safety Investment Information System and Dynamic Supervision Mechanism

#### 3.2.1. Safety Investment Information System

Previous studies have proposed safety information systems to improve general safety supervision via the effective utilization of information technology [25,26,46]. In this subsection, from the perspective of expense supervision, we propose a safety investment information system that can be defined as a safety investment information record and evaluation system that utilizes information technology. By adopting the safety investment information system, communication between the government and contractors could be strengthened, which could further support the government in implementing more dynamic supervision.

As an effective way of utilizing the wisdom of groups and proposing innovative ideas [47], several brainstorming seminars were organized to discuss the structure of safety investment information systems. In addition to our research team, the participants of these seminars also included professionals from the government, proprietors, construction supervising engineers and contractors. In the first seminar, professionals proposed the concept of a safety investment recording system for construction contractors, namely, a safety investment information system. In the second seminar, the participants’ responsibilities, the structure, and the information collection process of the information system are discussed and determined. In the final stage, the research team conducted several interviews with related experts in three typical Chinese cities located in northern, southeastern and central China. In these interviews, we surveyed the safety investment supervision procedures and measures of the local safety supervision departments to verify and amend the information system and make it suitable for national rollout.

(1) Participants and their responsibilities

**Figure 2:**
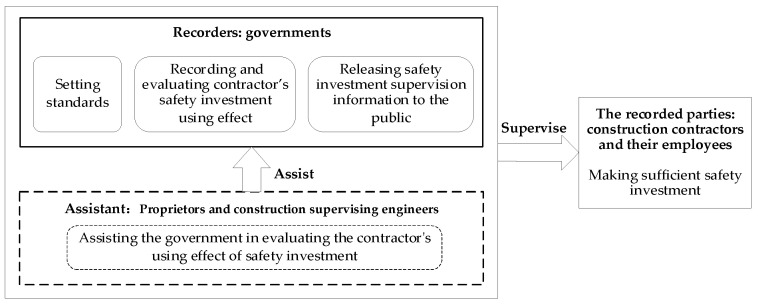
Relationships among participants and their duties.

There are four main participants in the safety investment information system: government, proprietors, construction supervising engineers, construction contractors and their employees. The four participants influenced and restricted each other, and their relationship is shown in Figure 2.

The government is more authoritative and plays a leading role in operating the safety investment information system. Therefore, we define the role of government as the recorder, aggregator, and publisher [26]. The government’s responsibilities are setting safety investment standards, releasing safety investment supervision information to the public, and recording and evaluating construction contractors’ safety investment use effect. Proprietors refer to enterprises or institutions responsible for ensuring the source of safety investment and allocating the safety investment to contractors on time according to the project nodes [48]. Construction supervising engineers are supervision professionals entrusted by proprietors as the representative to exercise supervision power [48]. In the safety investment information system, proprietors and construction supervising engineers are assistants who assist the government in restricting construction contractors’ illegal behaviors through daily supervision and evaluation. Construction contractors and their employees are supervised parties, and they shall comply with laws and regulations and make sufficient safety investment.

(2) Management process

**Figure 3:**
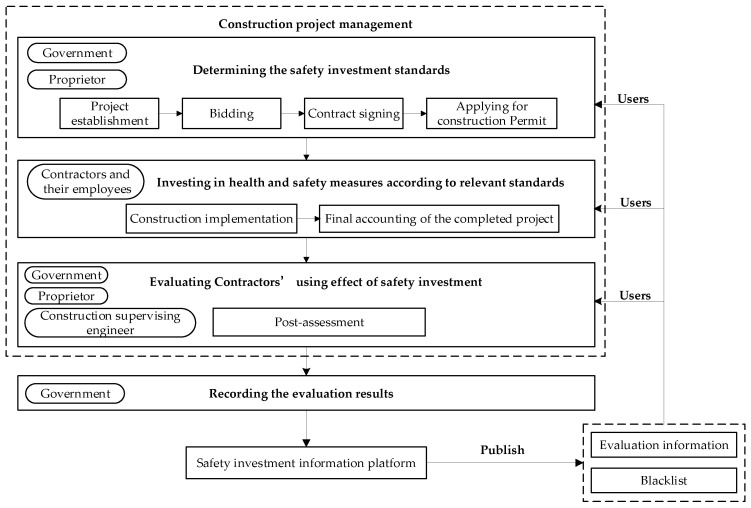
Structural diagram of the safety investment information system.

The foundation of the safety investment information system is real-time and reliable safety information. Figure 3 shows the structural diagram of the safety investment information system. In every construction project, the contractor’s use effect of safety investment at each stage is uploaded and recorded by the safety investment information system. The evaluation team composed of the government, the proprietor, and the construction supervising engineer evaluates the contractor’s use effect of safety investment through the information recorded by the safety investment information system. The evaluation results will accumulate continually over time. Based on the accumulated evaluation results, the contractors will automatically be rated by the safety investment information system, and contractors with poor performance in safety investment will be put on the blacklist, which will affect contractors’ business activities.

There are four types of users in the safety investment information system: the government, proprietors, construction supervising engineers, construction contractors and their employees. The government, proprietors, and construction supervision engineers can obtain the contractors’ historical safety performance from the information accumulated in the system and then implement more flexible supervision strategies. Simultaneously, as subcontracting is common in the construction industry, the main contractors also need to use the information system to acquire the subcontractors’ safety performance. For contractors’ employees, since the work environment is of great importance for job satisfaction [49] and an increase in safety investment can result in a better safe atmosphere of the work environment [33], they can survey information published by the government and choose a construction enterprise with good performance in safety investment as their employer to enjoy a better working environment.

#### 3.2.2. Dynamic Supervision Mechanism Based on the Safety Investment Information System

**Figure 4:**
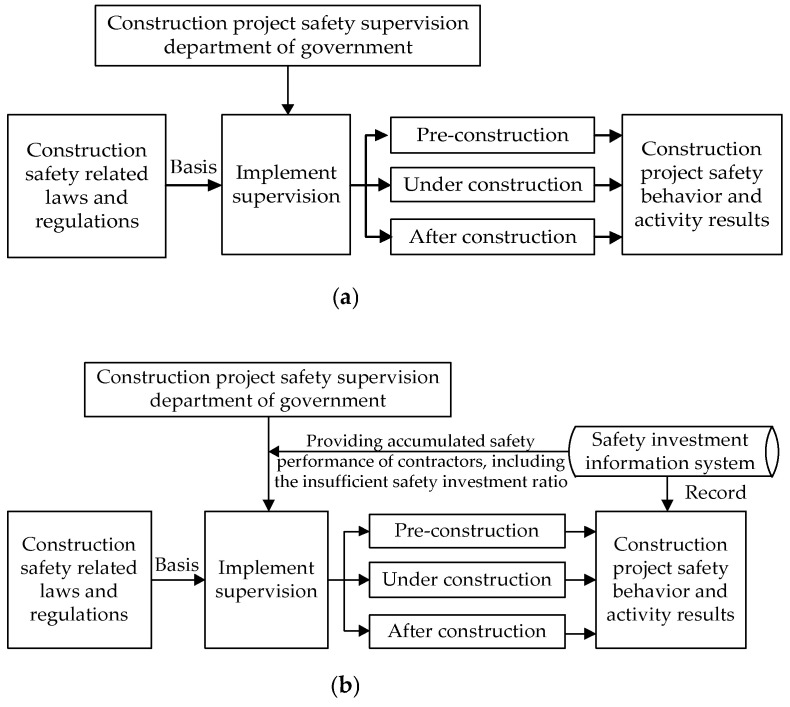
Structural diagram of supervision mechanism: (**a**) static supervision and (**b**) dynamic supervision.

Under the current construction safety investment supervision mode, the government inspects construction sites regularly. Once illegal behaviors (e.g., insufficient safety investment) of contractors are found, the government will impose punitive measures on contractors based on relevant laws and regulations. However, the information obtained by the government about safety production based on on-site inspections is delayed and partial [21]; therefore, the proposed supervision strategies based on these messages are relatively fixed and static and cannot match the actual safety production situation. This kind of supervision mode has been defined as a “static supervision mechanism” [50,51], and its structural diagram is shown in Figure 4a [52].

After adopting the safety investment information system, the information system can record contractors’ historical safety performance and the use effects of safety investment in the current project. The government can acquire information from the information system and then adjust its supervision measures in response to varying degrees of safety production situations in real time. The supervision strategies become more flexible and dynamic in this scenario. This kind of supervision mode has been defined as a “dynamic supervision mechanism” in existing research in other fields [11,19], and its structural diagram is shown in Figure 4b.

### 3.3. Multiplayer Evolutionary Game Model

In the process of safety investment supervision, the different demands and objectives of the government and contractors result in conflicts of interest; furthermore, under the dynamic supervision mechanism, the interactions between stakeholders will become much more complicated [11]. The government and contractors behave as bounded rational stakeholders, by observing and comparing payoffs with others, they adjust their strategies dynamically to maximize their interests [27]. The interactions among these stakeholders can be regarded as a dynamic game process. Evolutionary game theory is one of the most fruitful frameworks to study dynamic adaptation and learn in repeated games played by bounded rational players [53]. This process pays attention to the dynamics of strategic change [50]. Therefore, in this subsection, multiplayer evolutionary game theory was adopted to study the long-term decision-making process of stakeholders.

#### 3.3.1. Game Relationship Description and Assumptions

**Figure 5:**
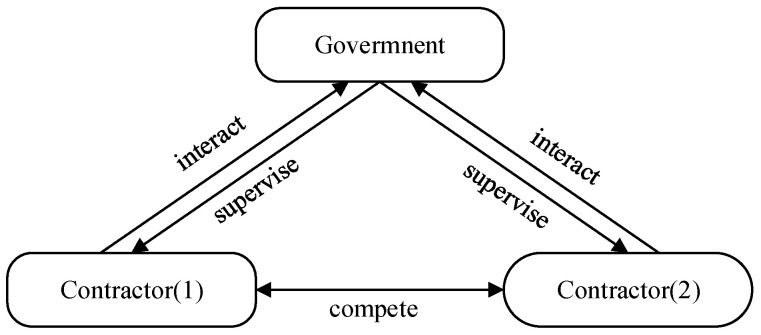
Multi-player game model of construction safety investment supervision.

Given the actual situation of construction safety investment supervision, when there are a number of construction contractors, different contractors will have different interests and demands between them under government supervision [11]. To simplify the research, we concentrate on interactions between the government and two competing contractors with different safety investment levels. The multiplayer game model of construction safety investment supervision is shown in Figure 5, and the relationship among the stakeholders is described as follows.

The government supervises contractors in relation to safety investment. Considering construction safety and supervision costs, the government will choose active supervision or negative supervision. Therefore, the government’s behavioral strategy space is (supervision, not supervision). With the purpose of pursuing maximum profit, the two competing contractors can choose to make sufficient safety investment or not. Therefore, their strategy space is (making sufficient safety investment, not making sufficient safety investment).

To maintain the objectivity and scientificity of the evolutionary game model, several assumptions are proposed as follows: In construction safety investment supervision, the government can be regarded as a bounded rational stakeholder who changes its strategies dynamically by observing and comparing payoffs with others [27]. Simultaneously, public interest theory points out that government regulation corrects the defects of market failure to protect the public interest [54,55]. Based on those popular understandings, we propose our first two assumptions to describe the government’s behavior target and simplify the relationship between government and contractors.

**Assumption** **1.** *The government has limited rationality and attaches great importance to the social benefits generated by projects.*

**Assumption** **2.** *The government’s supervisory ability is sufficiently strong. That is, if the government chooses to supervise, then a violation of the regulations by the contractor will be punished immediately.*

In the construction industry, construction contractors may unavoidably conduct a cost-safety trade-off in the bidding and construction stages because of limited resources [9]. Hence, contractors may minimize their costs of safety production for profit maximization [15]. Therefore, the contractors’ behavior target can be defined as follows.

**Assumption** **3.** *The two contractors are assumed to be economic men who make strategic choices according to the principle of profit maximization.*

#### 3.3.2. Parameters and Payoff Matrix

Based on the Chinese construction context, we propose the following parameters for the safety investment supervision process.

Assume that the government supervises the contractors’ construction safety investment with a probability *x* (0 ≤ *x* ≤ 1). The level of the supervision probability *x* represents the strength of safety investment supervision. *x* = 0 means that the government chooses not to supervise contractors’ safety investment, and *x* = 1 means that the government supervises the contractors in real time. In addition, the government needs to invest workforce and material resources in the supervision process, and these expenditures can be regarded as government safety supervision costs [15]. *C_g_* represents the safety supervision cost of the government.

Assume that the two contractors with competitive relations choose to make sufficient safety investment with a probability *y_i_* (0 ≤ *y* ≤ 1, *i* = 1, 2). Therefore, the probability of contractors does not make sufficient safety investment is (1 − *y_i_*). During the process of construction, *R_i_* (*i* = 1, 2) represents the normal revenue from regular safety production and *S_i_* (*i* = 1, 2) represents the standard safety investment required in laws and regulations. When contractors choose not to make sufficient safety investment, they can save safety cost but will undermine the social benefit. Assuming that contractors’ actual safety investment is *S_i_’* (*i* = 1, 2, *S_i_ > Si’*), the loss of overall social benefit caused by contractors’ insufficient safety investment is represented as *L_i_* (*i* = 1, 2).

Based on the Administrative Regulations on the Work Safety of Construction Projects [56], which was enacted by the State Council of the People’s Republic of China, contractors will be fined according to the degree of insufficient safety investment, and the penalty coefficient *k* is between 1.2 and 1.5. According to our investigation, contractors with a high degree of insufficient safety investment will have a higher penalty coefficient than contractors with a low degree of insufficient safety investment.

Therefore, the penalty (*P_i_*) for contractor (*i*) under different supervision mechanisms can be represented as follows.

- Static supervision mechanism

Under the static supervision mechanism adopted by the government in the present actual operation, contractors are fined according to the static penalty control strategy. When only one contractor (*i*) chooses not to obey laws and regulations and does not make sufficient safety investment, the penalty (*P_i_*) for contractor (*i*) is shown as follows:

(1) $$P_{i}=k_{i}\left(S_{i}-{S_{i}}^{\prime }\right)\;(1.2\leq k_{i}\leq 1.5,S_{i}>{S_{i}}^{\prime },i=1,\;2,\;(k_{1}-k_{2})[\left(S_{1}-{S_{1}}^{\prime }\right)-\left(S_{2}-{S_{2}}^{\prime }\right)]\geq 0)$$

- Dynamic supervision mechanism

Under the dynamic supervision mechanism, contractors are fined according to the dynamic penalty control strategy. With the use of the information system, the government can obtain instant information on the contractor′s safety investment use effectiveness, which enables the government to link the contractors′ penalty (Pi) to their insufficient safety investment probability (1−yi) recorded by the safety information system, as shown in Equation (2).

(2) $$P_{i}=k_{i}\left(S_{i}-{S_{i}}^{\prime }\right)\left(1-y_{i}\right)+k_{i}\left(S_{i}-{S_{i}}^{\prime }\right)(1.2\leq k_{i}\leq 1.5,S_{i}>{S_{i}}^{\prime },i=1,\;2,\;(k_{1}-k_{2})[\left(S_{1}-{S_{1}}^{\prime }\right)-\left(S_{2}-{S_{2}}^{\prime }\right)]\geq 0)$$

Furthermore, when both contractors choose to not obey laws and regulations and not make sufficient safety investment, if the government chooses to supervise, to act as a deterrent, the penalty (*P*) for contractors is shown as follows:

*P* = *P*_1_ + *P*_2_ (3)

The variables of the multiplayer game are shown in Table 1. According to the above assumptions, the payment matrix between the two contractors with competitive relations is shown in Table 2.

The government’s payoff matrix with the two contractors is shown in Table 3 below.

### 3.4. System Dynamics Simulation and Validation

#### 3.4.1. System Dynamics Model Settings

System dynamics is a quantitative simulation method to analyze information feedback mechanism, which is often used to study complex systems [11,57]. In previous studies, given the complex nonlinear relationship among multiplayer evolutionary game players, SD has been applied to simulate the stability of the equilibrium solution of multiplayer evolutionary games, such as resource allocation [58], public-private partnership projects [59], environmental regulation [60], and safety regulation [11]. Therefore, based on the stakeholders’ feedback behaviors in the evolutionary game model analyzed above, SD was applied to simulate and validate the evolutionary game process and analyze the implementation effect and equilibrium state of different supervision strategies. The model settings are INITIAL TIME = 0, FINAL TIME = 50, TIME STEP = 0.0078125, Unit for Time = Year, Integration Type = Euler.

#### 3.4.2. Validation

To validate the dynamic supervision mechanism’s effectiveness, we substitute the actual data of safety supervision in China into SD model for simulation. According to the China Statistical Yearbook on Construction [61], related laws and regulations in China, and surveys conducted with related experts in the construction industry, the initial values of external variables in the SD model are shown in Table 4 after pretreatment. The initial values of the three level variables are assumed to be *x* = *y*_1_ = *y*_2_ = 0.5.

The specific acquisition of the data is explained as follows: According to the China Statistical Yearbook on Construction [61], the contractors’ average normal revenue (*R)* from regular safety production accounts for 6.3% of the total project cost, therefore, we could assume *R*_1_ accounts for 8% and *R*_2_ accounts for 6% of the total project cost. The management regulations related to safety investment in Shanghai stipulate that the construction safety investment (*S*) accounts for 3% of the project cost [62]. Based on the Administrative Regulations on the Work Safety of Construction Projects [56], the penalty coefficient *k_i_* is between 1.2 and 1.5, and contractors with a high degree of insufficient safety investment will have a higher penalty coefficient than contractors with a low degree of insufficient safety investment. According to our investigation results of experts from government construction safety supervision authorities, *C_g_* approximately accounts for 2‰ of the total project cost, *L_i_* is approximately 1.2 times of the contractor’s degree of insufficient safety investment (S − S’). Therefore, we assume *S_1′_* accounts for 2.5% and *S_2′_* accounts for 2.8% of the total project cost, and we can reach the result that *L*_1_ accounts for 0.6% and *L*_2_ accounts for 0.24% of the total project cost. As the evolutionary game model and SD simulation focus on the proportional relationship between variables, therefore, to make the data easy to calculate, we regard *C_g_* as 1, after pretreatment, the initial values of external variables in the SD model are as in Table 4.

## 4. Results

### 4.1. Replicator Dynamics of the Multiple-Player Evolutionary Game Model

According to evolutionary game theory, replicator dynamics reflect the dynamic change direction of strategy proportion. In the process of the construction safety investment supervision, the replication dynamics can be used to reflect the dynamic strategy adjustment process of individuals. Therefore, the following replicator dynamics equation set can be used to represent the multiplayer evolutionary game of construction safety investment supervision (the details to solve the evolutionary game model and obtain the replicator dynamics equation set are presented in Appendix A):

(4) $$\left\{\begin{array}{ll} F(y_{1},y_{2},x)=\frac{dy_{1}}{dt} & =y_{1}(1-y_{1})(U_{y1}-U_{1-y1})\\ & =y_{1}(1-y_{1})[R_{1}-S_{1}-y_{2}(R_{1}-{S_{1}}^{\prime }-xP_{1})-(1-y_{2})(R_{1}-{S_{1}}^{\prime }-xP)]\\ & =y_{1}(1-y_{1})[y_{2}({S_{1}}^{\prime }-S_{1}+xP_{1})+(1-y_{2})({S_{1}}^{\prime }-S_{1}+xP)]\\ G(y_{1},y_{2},x)=\frac{dy_{2}}{dt} & =y_{2}(1-y_{2})(U_{y_{2}}-U_{1-y_{2}})\\ & =y_{2}(1-y_{2})[R_{2}-S_{2}-y_{1}(R_{2}-{S_{2}}^{\prime }-xP_{2})-(1-y_{1})(R_{2}-{S_{2}}^{\prime }-xP)]\\ & =y_{2}(1-y_{2})[y_{1}({S_{2}}^{\prime }-S_{2}+xP_{2})+(1-y_{1})({S_{2}}^{\prime }-S_{2}+xP)]\\ H(y_{1},y_{2},x)=\frac{dx}{dt} & =x(1-x)\left(U_{x}-U_{1-x}\right)\\ & =x(1-x)[y_{1}y_{2}(-C_{g})+y_{1}(1-y_{2})(P_{2}-C_{g})+(1-y_{1})y_{2}(P_{1}-C_{g})\\ & +(1-y_{1})(1-y_{2})(P-C_{g})] \end{array}\right\}$$

When equation set (4) is equal to 0, the game reaches a relatively stable equilibrium state. According to Friedman’s theory [63], by analyzing the determinant *Det* (*J*) and the sign of the trace value *Tr* (*J*) of the Jacobian matrix *Det* (*J*) at the equilibrium point, we can judge whether the equilibrium point is an evolutionarily stable strategy (ESS) equilibrium.

### 4.2. Stock and Flow Diagram of SD

**Figure 6:**
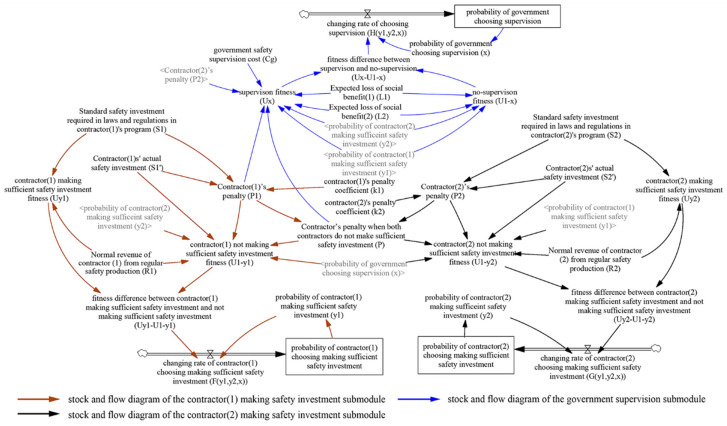
The stock and flow diagrams of evolutionary game SD model under static penalty control strategy.

**Figure 7:**
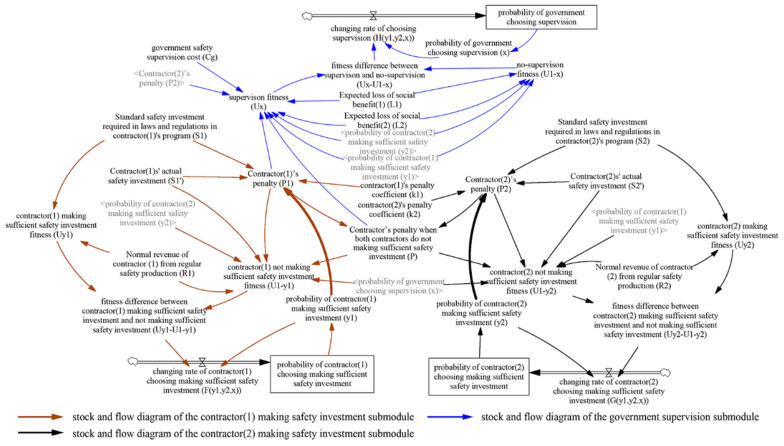
The stock and flow diagrams of evolutionary game SD model under dynamic penalty control strategy.

Vensim 6.4e software is used to establish the multiplayer evolutionary game SD model of construction safety investment supervision according to the above game assumptions and analysis (Section 3.3 and Section 4.1). Regarding these assumptions and analyses of the static and dynamic penalty control strategies, the stock and flow diagrams of the SD models are shown in Figure 6 and Figure 7, respectively.

In Figure 6 and Figure 7, the arrows indicate the stock and flow diagrams of the evolutionary game under the static and dynamic penalty control strategies, respectively. Under the dynamic penalty control strategy, the government can obtain information on contractors’ previous safety performance and then link the contractors’ penalty to the rate of their insufficient safety investment, as shown in Equation (2) above. Therefore, under this scenario, there are arrows between contractors’ penalty (*P*_1_, *P*_2_) and contractors’ not making sufficient safety investment probability ((1 − *y*_1_), (1 − *y*_2_)), which is reflected by the bold arrows in Figure 7.

The evolutionary game SD model under the static and dynamic penalty control strategies comprises three level variables, three rate variables, fifteen auxiliary variables, and eleven external variables. In the static scenario, the functional relationship among those variables is based on the dynamic replication equation of the above multiplayer evolutionary game model, namely, Equations (1), (3) and (4). In the dynamic scenario, the functional relationship among those variables is based on Equations (2)–(4). The meanings of these variables in the two scenarios are the same. Specifically, level variables represent the system’s accumulations, which involve the probability of the contractor (1) making a sufficient safety investment, the probability of the contractor (2) making a sufficient safety investment, and the probability of government choosing supervision. Rate variables represent the flow in the system caused by the decision-making process, which involve the changing rate of contractor (1) choosing to make a sufficient safety investment *(F(y*_1_*,y*_2_*,x))*, the changing rate of contractor (2) choosing to make a sufficient safety investment *(G(y*_1_*,y*_2_*,x))*, and the changing rate of government choosing supervision *(H(y*_1_*,y*_2_*,x))*. Auxiliary variables (i.e., contractor (1) making sufficient safety investment fitness) refer to transition variables by calculation. External variables are constants in the system and consistent with the values of variables in the payment matrix of the evolutionary game in Table 2 and Table 3. Simultaneously, there are three submodules in the SD model, and we use arrows of different colors to indicate the stock and flow diagrams of three different submodules, specifically, stock and flow diagram of the contractor (1) making safety investment submodule, stock and flow diagram of the contractor (2) making safety investment submodule and stock and flow diagram of the government supervision submodule. As the model has a large number of parameters, to avoid the crossing of arrows and clarify the information in the diagram, the parameters that have been defined in one submodule (dark text) are represented by shadow variables (gray text) in another submodule. The detailed SD model equations can be found in Appendix B and Appendix C, the nomenclature can be found in Appendix D.

### 4.3. Simulation Results Analysis

#### 4.3.1. Evolutionary Game Player’s Strategy Choices under Different Penalty Control Strategies

**Figure 8:**
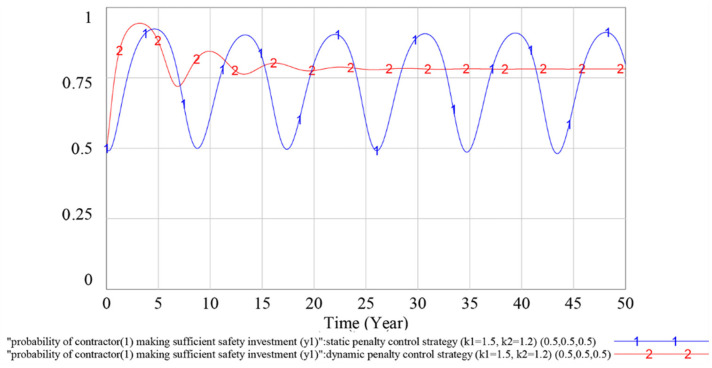
Influence of different penalty control strategies on contractor (1)’s strategy choices.

**Figure 9:**
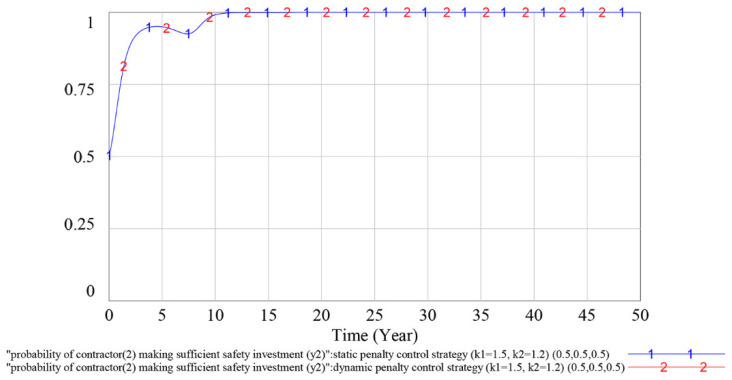
Influence of different penalty control strategies on contractor (2)’s strategy choices.

**Figure 10:**
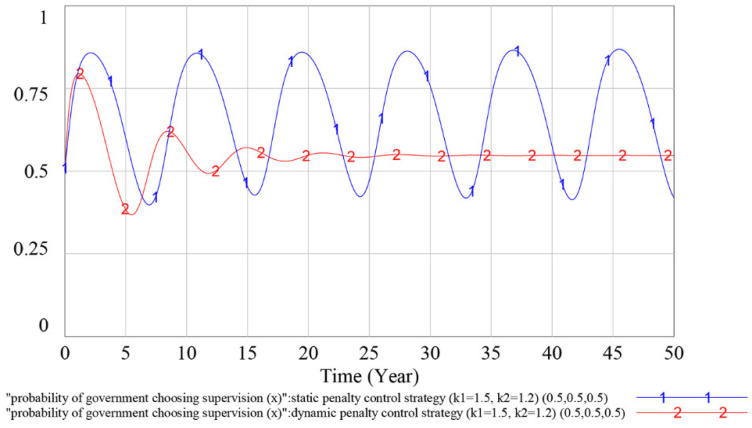
Influence of different penalty control strategies on the government’s strategy choices.

To verify the effectiveness of the dynamic penalty control strategy, we compare the simulation results of the evolutionary game SD model under the static penalty control strategy and the dynamic penalty control strategy in this subsection. The simulation results are shown in Figure 8, Figure 9 and Figure 10.

According to Figure 8, under the static penalty control strategy, contractor (1)’s strategy selections fluctuate periodically, indicating that the equilibrium point of the evolutionary game does not exist. Simultaneously, the dynamic penalty control strategy can suppress the fluctuation of contractor (1)’s strategy choices and make the game stable. According to Figure 9, as curve 1 and curve 2 overlap with each other, we can reach the result that the different penalty control strategies do not influence contractor (2)’s strategy choices. According to Figure 10, under the static penalty control strategy, the government’s strategy selections fluctuate periodically, indicating that the equilibrium point of the evolutionary game does not exist. Simultaneously, under the dynamic penalty control strategy, the fluctuation of the government’s strategy choices could be effectively restrained, thereby stabilizing the game.

In sum, we can reach the result that under the static penalty control strategy, the equilibrium point of the evolutionary game does not exist, while adopting the dynamic supervision strategy can effectively suppress the fluctuation of the game process and make the game stable. Simultaneously, because the probability at which the contractor (2) making a sufficient safety investment can reach a stable state of *y*_2_ = 1, which is much higher than that of contractor (1), and the profit of an insufficient safety investment for the contractor (2) is smaller than that of contractor (1), we find that the contractor with a small profit associated with an insufficient safety investment is more inclined to choose to obey the rules than the contractor with a large profit associated with an insufficient safety investment.

#### 4.3.2. Evolutionary Stable Equilibrium Analysis

**Figure 11:**
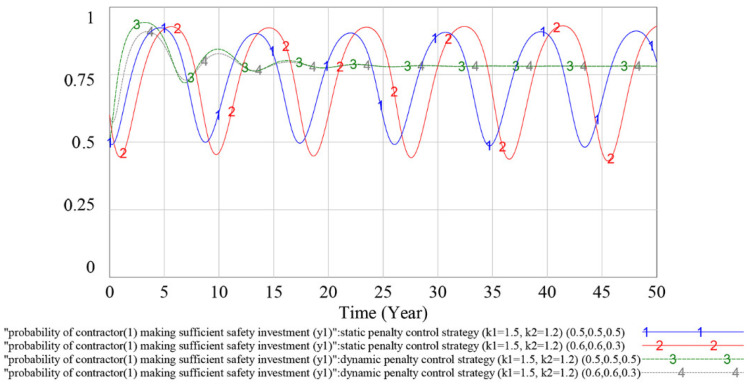
Influence of different initial values on contractor (1)’s strategy choices under different penalty control strategies.

**Figure 12:**
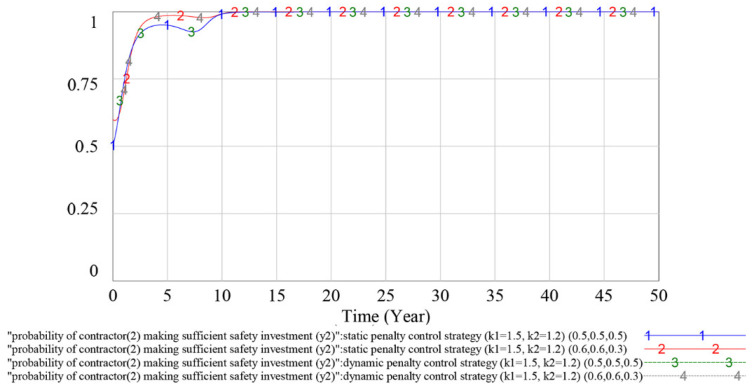
Influence of different initial values on contractor (2)’s strategy choices under different penalty control strategies.

**Figure 13:**
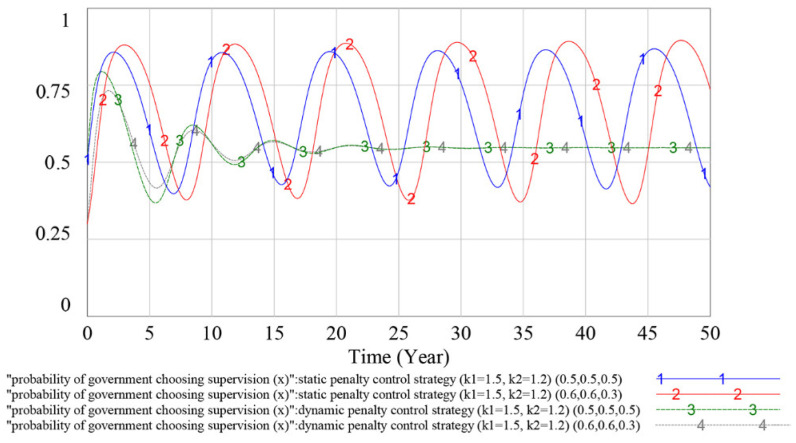
Influence of different initial values on the government’s strategy choices under different penalty control strategies.

To analyze whether the game has an evolutionary stability strategy under the static and dynamic penalty control strategies, this subsection simulates the different initial values of the parties to the game. If the game players choose different initial values and the game finally evolves to the same result, then the game has an ESS under this penalty control strategy. The simulation results are shown in Figure 11, Figure 12 and Figure 13 below.

In Figure 11, Figure 12 and Figure 13, curves 1 and 2 indicate the evolutionary game process under the static penalty control strategy (*k*_1_ = 1.5, *k*_2_ = 1.2), and the initial values of the game are (*y*_1_, *y*_2_, *x*) = (0.5, 0.5, 0.5) and (*y*_1_, *y*_2_, *x*) = (0.6, 0.6, 0.3), respectively; and curves 3 and 4 indicate the evolutionary game process under the dynamic penalty control strategy (*k*_1_ = 1.5, *k*_2_ = 1.2), and the initial values of the game are (*y*_1_, *y*_2_, *x*) = (0.5, 0.5, 0.5) and (*y*_1_, *y*_2_, *x*) = (0.6, 0.6, 0.3), respectively.

According to the above simulation results, under the dynamic penalty control strategy, the change in the initial value (*y*_1_, *y*_2_, *x*) does not have much impact on the dynamics and equilibrium point of the game evolution process. With the increase in game times, the strategy choices of game players gradually stabilize in a certain equilibrium state. Under the static penalty control strategy, different initial values have different effects on the strategy evolution process of the contractor (1) and government, and the amplitude and frequency of volatility will change in the evolution process with different game initial values.

Therefore, based on the above simulation results, the game has no ESS under the static penalty control strategy. In addition, compared with the static scenario, using the dynamic penalty control strategy can make the game reach an ESS, and this result could validate the effectiveness of the dynamic penalty control strategy.

#### 4.3.3. Optimization of the Dynamic Penalty Control Strategy

For the government, the main purpose of formulating penalty control strategies is not to punish but to eliminate the illegal operation of contractors. From a construction industry aspect, the above analysis shows that the dynamic penalty control strategy can effectively suppress the fluctuation of the players’ strategy choices in the safety investment supervision evolutionary game, which further improves the efficiency and sustainability of government supervision. However, from a construction project management aspect, the dynamic penalty control strategy has some limitations, e.g., the construction project cycle is generally 1–2 years but the abovementioned dynamic penalty strategy will obviously suppress the fluctuation of the game process after 5–10 years, which is much longer than 2 years. Simultaneously, when contractor (1)’s game process reaches the stable point, *y_1_* is approximately 0.80, meaning that contractor (1) owes a 20% probability of not making a sufficient safety investment.

Many studies have proven that correlating penalties with players’ unlawful behavior probability and supervision probability can optimize the penalty control strategy [11,19,64]. Therefore, the optimized dynamic penalty control strategy is proposed to make the game process reach an ideal ESS in advance, which will further improve its applicability in construction project management. The formula is shown as follows.

(5) $$P_{i}=k_{i}\left(S_{i}-{S_{i}}^{\prime }\right)\left(1-y_{i}\right)+\;k_{i}\left(S_{i}-{S_{i}}^{\prime }\right)+\frac{\left(S_{i}-{S_{i}}^{\prime }\right)}{x}(1.2\leq \;k_{i}\leq 1.5,S_{i}>{S_{i}}^{\prime },i=1,2,\;(k_{1}-k_{2})[\left(S_{1}-{S_{1}}^{\prime }\right)-\left(S_{2}-{S_{2}}^{\prime }\right)]\geq 0)$$

**Figure 14:**
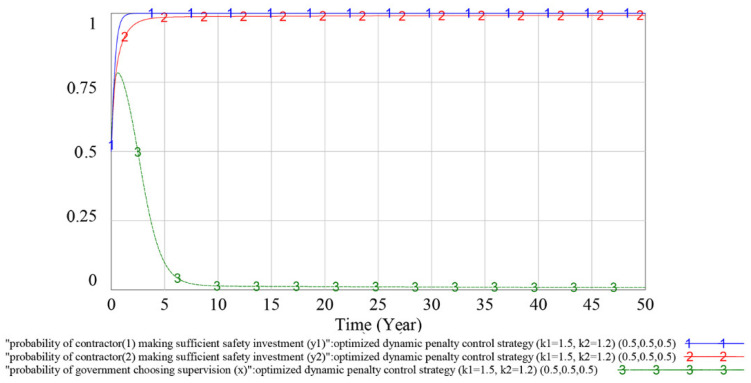
Simulation results under optimized dynamic penalty control strategy (initial value (*y_1_* = *y_2_* = *x* = 0.5)).

**Figure 15:**
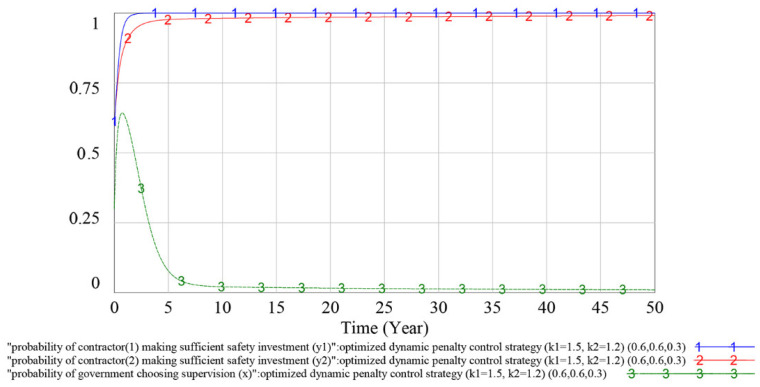
Simulation results under optimized dynamic penalty control strategy (initial value (*y_1_* = 0.6, *y_2_* = 0.6, *x* = 0.3)).

When using the optimized dynamic penalty control strategy, the simulation results of the game process are shown in Figure 14 and Figure 15.

In Figure 14, curves 1, 2 and 3 indicate the evolutionary game process of contractor (1), contractor (2) and government under the optimized dynamic penalty control strategy (*k*_1_ = 1.5, *k*_2_ = 1.2), respectively, and the initial value of the game is (*y*_1_, *y*_2_, *x*) = (0.5, 0.5, 0.5). A comparison of Figure 14 with Figure 11, Figure 12 and Figure 13 shows that under the same penalty coefficient, the stability of the evolutionary game process is further improved after correlating penalties with players’ unlawful behavior probability and supervision probability, and the players’ strategy selection probability quickly reaches a stable ideal value.

To analyze whether the game has an evolutionary stability strategy under the optimized dynamic penalty control strategy, we adjust the initial value of variables to (*y*_1_, *y*_2_, *x*) = (0.6, 0.6, 0.3), and the simulation result is shown in Figure 15. The simulation result shows that the change in the initial value (*y*_1_, *y*_2_, *x*) does not have much impact on the dynamics and equilibrium point of the game evolution process. This finding reflects that the optimized penalty control strategy can make the game process reach an ideal ESS in advance in which contractors could nearly choose to make a sufficient safety investment as their optimal strategy, and the effectiveness of the optimized dynamic penalty control strategy has been validated.

## 5. Discussion

As shown in Figure 8, Figure 9 and Figure 10, under the static penalty control strategy, the players’ strategy selection fluctuates periodically. This finding is consistent with previous studies [11,57] and shows that in the safety supervision process, when considering different interest demands among multiple participants, the strategy selection of participants will fluctuate and ESS does not occur in the game process. This result reflects problems associated with the current static supervision mode of China. When the contractor’s safety investment is insufficient and accidents frequently occur on the construction site, the government will enforce severe punishment by increasing the on-site supervision frequency, which will immediately increase the contractors’ safety investment rate. However, as the safety atmosphere in the construction market improves, to reduce the cost of supervision, the government will gradually slacken supervision to the contractors’ safety investment level and the contractors will gradually reduce the safety investment rate, which will in turn aggravate the safety problems at the construction site. The volatility that occurs during the game and the repetition of contractors’ inadequate safety investment can easily lead to incorrect predictions by the government about the implementation of the strategy or introduce doubts about the reasonable applicability of existing safety investment regulatory policies and thus lead to incorrect strategy selection.

By adopting the dynamic supervision mechanism, the government can obtain information on contractors’ safety investment use effectiveness, which will enable the government to correlate its penalty strategy (P) with contractors’ unlawful behavior probability ($1-y_{i}$). The simulation results in Section 4 show that under this scenario, the fluctuation of the evolutionary game can be effectively restrained and the strategic choices of game players gradually stabilize in a certain equilibrium state strategy. The result is consistent with the argument proposed by Wang et al. (2011) in which the dynamic penalty was suggested as effective for evolutionary game equilibrium stabilization [51]. From the perspective of the safety information system, our research is consistent with the finding of Pi et al. (2019), who revealed that by applying the safety information system, the contractors’ rule-breaking behavior would be restrained by their credit rating [26]. Moreover, in contrast to Pi et al.’s (2019) research, where a two-player game between the government and contractors is developed to verify the information system’s effectiveness, we take the complex interactions between multiple contractors into consideration and build the multiplayer game to describe players’ strategy selection process, which is more consistent with the real operation of the construction industry.

As shown in Figure 11, Figure 12 and Figure 13, under the dynamic penalty control strategy, the evolutionary game reaches an ESS in 10–15 years. However, the construction project cycle is generally 1–2 years, which is much shorter than 10–15 years; therefore, in terms of safety investment supervision, the dynamic penalty control strategy is more applicable at the industry level than the project level. As shown in Figure 14 and Figure 15, the optimized dynamic penalty control strategy can make the game process reach an ideal ESS in advance; therefore, from the aspect of construction project management, the optimized scenario can improve the applicability of the dynamic penalty control strategy.

Simultaneously, according to the analysis in Section 4 and regardless of the static supervision mode or dynamic supervision mode, contractor (2) could nearly choose to make a sufficient safety investment as its optimal strategy and the profit of insufficient safety investment for contractor (2) is much smaller than that for contractor (1). Therefore, the contractor with a small profit associated with an insufficient safety investment is more inclined to choose to obey the rules than the contractor with a large profit associated with an insufficient safety investment, which shows that the contractor with a small illegal return belongs to the risk conservative type in the model and the contractor with a large illegal return belongs to the risk preference type. This finding supports the arguments put forward by Cheung and Zhuang, who indicated that competition could influence the company’s threshold for risk [65].

Furthermore, based on the basic points of public interest theory [54,55], we assume that the government attaches great importance to social benefits and that the government’s supervision ability is strong enough, which simplifies the relationship between the government and contractors. According to the simulation results in Figure 10, Figure 13 and Figure 15, the government’s strategy selection can be influenced by the government’s penalty on contractors and contractors’ rule-breaking behavior, which is in line with the previous study [19,66]. However, when considering rent-seeking between the government and contractors, Feng et al. raised the argument that rent-seeking weakens the regulation utility of safety supervision [67] and Chen et al. found that government supervision probability is influenced by penalties and bribery [68]. Therefore, rent-seeking can make relationships between the government and contractors much more complicated and could be included in a further study.

According to the above results and analysis, the following management recommendations are proposed. (1) For governments, to eliminate the information gap between governments and contractors during construction safety investment supervision, governments need to pay attention to the construction of safe investment information systems. In addition, the government could adopt the dynamic penalty control strategy proposed in this research to cope with the dynamic nature of construction jobsites. Moreover, according to the above analysis, under the dynamic penalty control strategy, which enables the government to correlate its penalty strategy with contractors’ unlawful behavior probability, contractors will proactively make sufficient safety investment in accordance with laws and regulations in a long-term and stable manner. Therefore, governments could save supervision costs and improve supervision effectiveness. (2) For contractors, to achieve long-term interests, they should proactively make sufficient safety investment to build goodwill to gain the trust of governments and business partners. Simultaneously, with a sufficient safety investment, not only could the contractors adopt basic safety protection measures that are mandated by laws and regulations but they could also optimize the structure of fund use, such as increasing the investment in research of safety protection measures and strengthening safety education on construction workers, which could further increase the workers’ wellbeing in construction job sites.

Although the model in this research is established according to the Chinese construction context, it can be extrapolated to other geographical contexts. First, as resources are limited, construction contractors conduct a cost-safety trade-off unavoidably in construction process, which will result in insufficient safety investment and further undermine public interests. Simultaneously, as government regulation protects the public interest [54,55], in most countries, governments supervise contractors’ safety investment and penalize them for their illegal behaviors [9,65]. Hence, the economic interest relationship between safety supervision stakeholders discussed in this research also exists in many other countries. Second, in recent years, numerous researchers in other developing and developed countries have pointed out the need to improve supervision efficiency by narrowing the information gap between construction stakeholders [21,69,70]; thus, the safety investment information system proposed in our research could help to solve information gap issues and improve supervision efficiency in other countries. Therefore, to some extent, the safety investment information system and theoretical model established in this research can be extrapolated to analyze other safety supervision systems.

## 6. Conclusions

Safety investment and safety supervision play a pivotal role in improving occupational health and safety in the construction industry. Existing studies mainly focus on two aspects, the first is the safety supervision decision-making optimization, and the second is the establishment of information systems to strengthen the information communication between the safety supervision stakeholders. However, how the information system influences the stakeholders’ decision-making in the long-term safety supervision process remains largely unknown. The present study has firstly developed a dynamic safety investment supervision mechanism based on establishing a safety investment information system to fill the information gap between the government and contractors. Then, evolutionary game theory and SD were used to describe and analyze the decision-making process of stakeholders under the dynamic supervision mechanism based on information system. The principal conclusions from this study are as follows:

(1) The proposed safety investment information system could contribute to eliminating the information gap between the government and contractors and facilitate the transition of the safety investment supervision mode from static to dynamic.
(2) Under the static penalty control strategy, the three stakeholders’ strategy selections fluctuate periodically and an ESS does not occur in the evolutionary game play.
(3) When using the dynamic penalty control strategy that correlates penalties with contractors’ unlawful behavior probability, the fluctuation of the stakeholders’ strategy choices can be effectively suppressed, and the game reaches an ESS at approximately 10-15 years after the game starts.
(4) Under the optimized scenario of the dynamic penalty control strategy, which further correlates penalties with the government supervision probability, not only is the fluctuation of stakeholders’ strategy choices effectively suppressed but the game process can also reach an ideal ESS in advance. In this scenario, the contractors could nearly choose to make a sufficient safety investment as their optimal strategy approximately 1–2 years after the game starts.
(5) Under the dynamic supervision mechanism based on the safety investment information system, as the probability of contractor making sufficient safety investment and the probability of government supervision can reach a stable ideal value in the game process, the dynamic supervision mechanism’s effectiveness in improving supervision efficiency is validated.

This study has several theoretical and practical implications. From theoretical aspects, our research results verify the effectiveness of dynamic supervision strategy in improving supervision efficiency, which is in line with previous researches in other industries. Meanwhile, the decision-making interactions of the multiple stakeholders were quantitatively analyzed to ascertain the stakeholders’ behavior characteristics under the dynamic supervision mechanism based on the information system. Furthermore, this study combined evolutionary game theory with SD to simulate the dynamic interactions among multiple stakeholders, and has achieved good results, which provides an efficient way to addresses complex dynamic problems. From practical aspects, first, the safety investment information system proposed in this study could promote information communication among supervision stakeholders and further improve their ability to cope with the dynamic nature of construction jobsites. Second, this study developed a dynamic supervision mechanism that could help to improve supervision efficiency and further improve construction safety performance in practice. Third, due to the advantages of the dynamic supervision mechanism, this study could promote the transformation of government regulatory thinking and provide a reference for the government’s specific policy-making. 

Nevertheless, there are still some limitations in our research. First, this study only considers the game between the government and contractors and does not include other stakeholders (e.g., proprietors and construction supervising engineers), the interactions with these stakeholders may influence the government’s and contractors’ strategy choices. Moreover, the dynamic safety investment supervision mechanism proposed in this study is based on the safety investment information system. However, this study only proposes the conceptual framework of the information system, as for the details of the information system, such as the indicators to evaluate contractors’ safety performance and unlawful behavior probability as well as the criterion to grade the contractors, need to be further addressed in future studies.

## Figures and Tables

**Table 1:** Meanings of the variables in the multiplayer game.

Symbols	Description of Symbols	Notes
*x*	Probability of government supervision	0 ≤ *x* ≤ 1
*y*_1_	Probability of contractor (1) making sufficient safety investment	0 ≤ *y*_1_ ≤ 1
*y*_2_	Probability of contractor (2) making sufficient safety investment	0 ≤ *y*_2_ ≤ 1
* C_g_*	Cost of safety supervision to the government	* C_g_* > 0
*R_i_*	Normal revenue from regular safety production	*R_i_* > 0, *i* = 1, 2
*S_i_*	Standard safety investment required in laws and regulations	*S_i_* > 0, *i* = 1, 2
* S_i_’ *	Contractors’ actual safety investment	* S_i_’ * > 0, *i* = 1, 2
*L_i_*	Expected loss of overall social benefit as a result of insufficient safety investment	*L_i_* > 0, *i* = 1, 2
*P_i_*	Contractor’s penalty when only one contractor does not make sufficient safety investment	*P_i_* > 0, *i* = 1, 2
*P*	Contractor’s penalty when two contractors do not make sufficient safety investment	*P* > 0
*k_i_*	Penalty coefficient	1.2 *≤ k_i_* *≤* 1.5 ^1^, *i* = 1, 2

^1^ Data source: The Administrative Regulations on the Work Safety of Construction Projects enacted by the State Council of the People’s Republic of China.

**Table 2:** Payoff matrix between the two competing contractors.

Strategy of Contractor (1)	Strategy of Contractor (2)	
	Making Sufficient Safety Investment (*y*_2_)	Not Making Sufficient Safety Investment (1 − *y*_2_)
Making sufficient safety investment (*y*_1_)	$R_{1}-S_{1},R_{2}-S_{2}$	$R_{1}-S_{1},R_{2}-{S_{2}}^{\prime }-xP_{2}$
Not making sufficient safety investment (1 − *y*_1_)	$R_{1}-{S_{1}}^{\prime }-xP_{1},R_{2}-S_{2}$	$R_{1}-{S_{1}}^{\prime }-xP,R_{2}-{S_{2}}^{\prime }-xP$

**Table 3:** Payoff matrix of the government supervision departments with the two contractors.

Strategy of Contractor	Payoff of Government	
	Supervise (*x*)	Not Supervise (1 − *x*)
Contractor (1) makes sufficient safety investment and contractor (2) makes sufficient safety investment, (*y*_1_, *y*_2_)	−*C_g_*	0
Contractor (1) makes sufficient safety investment and contractor (2) does not make sufficient safety investment, (*y*_1_, 1 − *y*_2_)	P2−Cg−L2	−*L_2_*
Contractor (1) does not make sufficient safety investment and contractor (2) makes sufficient safety investment, (1 − *y*_1_, *y*_2_)	P1−Cg−L1	−*L_1_*
Contractor (1) does not make sufficient safety investment and contractor (2) does not make sufficient safety investment, (1 − *y*_1_, 1 − *y*_2_)	P−Cg−L1−L2	−*L_1_* − *L_2_*

**Table 4:** The initial values of external variables in the SD model.

Symbols	*C_g_*	*R*_1_	*R*_2_	*S*_1_	*S*_2_	*S*_1_′	*S*_2_′	*L*_1_	*L*_2_	*K_1_*	*K_2_*
Initial values	1	40	30	15	15	12.5	14	3	1.2	1.5	1.2

## Appendix A

In Section 4.1, the details to obtain the replicated dynamic equation set are shown as follows:

Contractor (1)’s making sufficient safety investment fitness (*U_y_*_1_) and not making sufficient safety investment fitness (*U*_1 − *y*1_) can be obtained as follows, respectively:

(A1) $$U_{y_{1}}=y_{2}(R_{1}-S_{1})+(1-y_{2})(R_{1}-S_{1})=R_{1}-S_{1}$$

(A2) $$U_{\left(1-y_{1}\right)}=y_{2}(R_{1}-{S_{1}}^{\prime }-xP_{1})+(1-y_{2})(R_{1}-{S_{1}}^{\prime }-xP)$$

Based on Equations (A1) and (A2), the average fitness of contractor (1) *U_y_*_1, 1 − *y*1_ is shown below:

(A3) $${\bar{U}}_{y_{1},1-y_{1}}=y_{1}U_{y_{1}}+(1-y_{1})U_{1-y_{1}}$$

Similarly, contractor (2)’s making sufficient safety investment fitness (*U_y_*_2_) and not making sufficient safety investment fitness (*U*_1 − *y*2_) can be obtained as follows, respectively:

(A4) $$U_{y_{2}}=y_{1}(R_{2}-S_{2})+(1-y_{1})(R_{2}-S_{2})=R_{2}-S_{2}$$

(A5) $$U_{\left(1-y_{2}\right)}=y_{1}(R_{2}-{S_{2}}^{\prime }-xP_{2})+(1-y_{1})(R_{2}-{S_{2}}^{\prime }-xP)$$

Based on Equations (A4) and (A5), the average fitness of contractor (2) *U_y_*_2__, 1 *− y*2_ is shown below:

(A6) $${\bar{U}}_{y_{2},1-y_{2}}=y_{2}U_{y_{2}}+(1-y_{2})U_{1-y_{2}}$$

Similarly, the government’s fitness of adopting supervision strategy and choosing the strategy of not to supervise, namely, *U_x_* and *U*_1 − *x*_, are obtained as follows, respectively:

(A7) $$\begin{array}{c} U_{x}=y_{1}y_{2}(-C_{g})+y_{1}(1-y_{2})(P_{2}-C_{g}-L_{2})+(1-y_{1})y_{2}(P_{1}-C_{g}-L_{1})\\ +(1-y_{1})(1-y_{2})(P-C_{g}-L_{1}-L_{2}) \end{array}$$

(A8) $$U_{1-x}=y_{1}(1-y_{2})(-L_{2})+(1-y_{1})y_{2}(-L_{1})+(1-y_{1})(1-y_{2})(-L_{1}-L_{2})$$

Based on Equations (A7) and (A8), the average fitness of government *U_x_*_, 1 − *x*_ is shown below:

(A9) $${\bar{U}}_{x,1-x}=xU_{x}+(1-x)U_{1-x}$$

In the process of replicator dynamics game, the rate of dynamic changes depends on the speed of learning or imitation, the change rate of *y*_1_ is shown as follows:

(A10) $$\frac{dy_{1}}{dt}{=y_{1}(}_{U_{y_{1}}}-{\bar{U}}_{y_{1},1-y_{1}})=y_{1}[U_{y_{1}}\;-y_{1}U_{y_{1}}\;-(1-y_{1})U_{1-y_{1}}]\;=\;y_{1}(1\;-y_{1})(U_{y_{1}}\;-U_{1-y_{1}})$$

Define $F(y_{1},y_{2},x)=\frac{dy_{1}}{dt}$ and bring Equations (A1) and (A2) into Equation (A10) and then obtain the contractor (1)’s replicated dynamic Equation (A11) as follows:

(A11) $$\begin{array}{c} F(y_{1},y_{2},x)=\frac{dy_{1}}{dt}=y_{1}(1-y_{1})[R_{1}-S_{1}-y_{2}(R_{1}-{S_{1}}^{\prime }-xP_{1})-(1-y_{2})(R_{1}-{S_{1}}^{\prime }-xP)]\\ \;=y_{1}(1-y_{1})[y_{2}({S_{1}}^{\prime }-S_{1}+xP_{1})+(1-y_{2})({S_{1}}^{\prime }-S_{1}+xP)] \end{array}$$

Similarly, make $G(y_{1},y_{2},x)=\frac{dy_{2}}{dt}$ and $H(y_{1},y_{2},x)=\frac{dx}{dt}$, the replicated dynamic equation of contractor (2) and government are obtained as follows, respectively:

(A12) $$\begin{array}{c} G(y_{1},y_{2},x)=\frac{dy_{2}}{dt}=y_{2}(1-y_{2})[R_{2}-S_{2}-y_{1}(R_{2}-{S_{2}}^{\prime }-xP_{2})-(1-y_{1})(R_{2}-{S_{2}}^{\prime }-xP)]\\ \;=y_{2}(1-y_{2})[y_{1}({S_{2}}^{\prime }-S_{2}+xP_{2})+(1-y_{1})({S_{2}}^{\prime }-S_{2}+xP)] \end{array}$$

(A13) $$\begin{array}{c} H(y_{1},y_{2},x)=\frac{dx}{dt}=x(1-x)[y_{1}y_{2}(-C_{g})+y_{1}(1-y_{2})(P_{2}-C_{g})+(1-y_{1})y_{2}(P_{1}-C_{g})\\ +(1-y_{1})(1-y_{2})(P-C_{g})] \end{array}$$

## Appendix B

The model equations of Figure 6.

Main equations of the contractor (1) making safety investment submodule:

(1) “contractor (1) making sufficient safety investment fitness (*U_y_*_1_)” = “Normal revenue of contractor (1) from regular safety production (*R*_1_)”—“Standard safety investment required in laws and regulations in contractor (1)’s program (*S*_1_)”
(2) “contractor (1) not making sufficient safety investment fitness (*U*_1−*y*1_)” = “probability of contractor (2) making sufficient safety investment (*y_2_*)” * (“Normal revenue of contractor (1) from regular safety production (*R*_1_)”—“Contractor (1)’s actual safety investment (*S*_1_’)”—“government supervision ratio (*x*)” * “Contractor (1)’s penalty (*P*_1_)”) + (1-”probability of contractor (2) making sufficient safety investment (y2)”) * (“Normal revenue of contractor (1) from regular safety production (*R*_1_)”—“Contractor (1)’s actual safety investment (*S*_1_’)”—“government supervision ratio (*x*)” * “Contractor’s penalty when both contractors do not make sufficient safety investment (*P*)”)
(3) “Contractor (1)’s penalty (*P*_1_)” = “penalty coefficient (*k*)” * (“Standard safety investment required in laws and regulations in contractor (1)’s program (*S*_1_)”—“Contractor (1)’s actual safety investment (*S*_1_′)”)
(4) “Contractor’s penalty when both contractors do not make sufficient safety investment (*P*)” = “Contractor (2)’s penalty (*P*_2_)” + “Contractor (1)’s penalty (*P*_1_)”
(5) “fitness difference between contractor (1) making sufficient safety investment and not making sufficient safety investment (*U_y_*_1_ − *U*_1−*y*1_)” = “contractor (1) making sufficient safety investment fitness (*U_y_*_1_)”—“contractor (1) not making sufficient safety investment fitness (*U*_1−*y*1_)”
(6) “changing rate of contractor (1) choosing making sufficient safety investment (*F* (*y*_1_, *y*_2_, *x*))” = “probability of contractor (1) making sufficient safety investment (*y*_1_)” * (1-”probability of contractor (1) making sufficient safety investment (*y*_1_)”) * “fitness difference between contractor (1) making sufficient safety investment and not making sufficient safety investment (*U_y_*_1_ − *U*_1−_*y*_1_)”

Main equations of the contractor (2) making safety investment submodule:

(7) “contractor (2) making sufficient safety investment fitness (*U_y_*_2_)” = “Normal revenue of contractor (2) from regular safety production (*R*_2_)”—“Standard safety investment required in laws and regulations in contractor (2)’s program (*S*_2_)”
(8) “contractor (2) not making sufficient safety investment fitness (*U*_1−y2_)” = “probability of contractor (1) making sufficient safety investment (*y*_1_)” * (“Normal revenue of contractor (2) from regular safety production (*R*_2_)”—“Contractor (2)’s actual safety investment (*S*_2_’)”—“government supervision ratio (*x*)” * “Contractor (2)’s penalty (*P*_2_)”) + (1-”probability of contractor (1) making sufficient safety investment (*y*_1_)”) * (“Normal revenue of contractor (2) from regular safety production (*R*_2_)”—“Contractor (2)’s actual safety investment (*S*_2_’)”—“government supervision ratio (*x*)” * “Contractor’s penalty when both contractors do not make sufficient safety investment (*P*)”)
(9) “Contractor (2)’s penalty (*P*_2_)” = “penalty coefficient (*k*)” * (“Standard safety investment required in laws and regulations in contractor (2)’s program (*S*_2_)”—“Contractor (2)’s actual safety investment (*S*_2_′)”)
(10) “Contractor’s penalty when both contractors do not make sufficient safety investment (*P*)” = “Contractor (2)’s penalty (*P*_2_)” + “Contractor (1)’s penalty (*P*_1_)”
(11) “fitness difference between contractor (2) making sufficient safety investment and not making sufficient safety investment (*U*_y2_ − *U*_1−y2_)” = “contractor (2) making sufficient safety investment fitness (*U*_y2_)”—“contractor (2) not making sufficient safety investment fitness (*U*_1-y2_)”
(12) “changing rate of contractor (2) choosing making sufficient safety investment (*G* (*y*_1_, *y*_2_, *x*))” = “probability of contractor (2) making sufficient safety investment (*y_2_*)” * (1-”contractor (2) making sufficient safety investment ratio (*y*_2_)”) * “fitness difference between contractor (2) making sufficient safety investment and not making sufficient safety investment (*U*_y2_ − *U*_1−y2_)”

Main equations of the government supervision submodule:

(13) “supervision fitness (*U_x_*)” = “probability of contractor (1) making sufficient safety investment (*y*_1_)” * “probability of contractor (2) making sufficient safety investment (*y_2_*)” * (-”government safety supervision cost (*C_g_*)”) + “probability of contractor (1) making sufficient safety investment (*y*_1_)” * (1-”probability of contractor (2) making sufficient safety investment (*y_2_*)”) * (“Contractor (2)’s penalty (*P*_2_)”—“government safety supervision cost (*C_g_*)”—“Expected loss of social benefit (2) (*L*_2_)”) + (1-”probability of contractor (1) making sufficient safety investment (*y*_1_)”) * “probability of contractor (2) making sufficient safety investment (*y_2_*)” * (“Contractor (1)’s penalty (*P*_1_)”—“government safety supervision cost (*C_g_*)”—“Expected loss of social benefit(1) (*L*_1_)”) + (1-”probability of contractor (1) making sufficient safety investment (*y*_1_)”) * (1-”probability of contractor (2) making sufficient safety investment (*y_2_*)”) * (“Contractor’s penalty when both contractors do not make sufficient safety investment (*P*)”—“government safety supervision cost (*C_g_*)”—“Expected loss of social benefit (1) (*L*_1_)”—“Expected loss of social benefit (2) (*L*_2_)”)
(14) “no-supervision fitness (*U*_1-*x*_)” = “probability of contractor (1) making sufficient safety investment (*y*_1_)” * (1-”probability of contractor (2) making sufficient safety investment (*y_2_*)”) * (-”Expected loss of social benefit (2) (*L*_2_)”) + (1-”probability of contractor (1) making sufficient safety investment (*y*_1_)”) * “probability of contractor (2) making sufficient safety investment (*y_2_*)” * (-”Expected loss of social benefit (1) (*L*_1_)”) + (1-”probability of contractor (1) making sufficient safety investment (*y*_1_)”) * (1-”probability of contractor (2) making sufficient safety investment (*y_2_*)”) * (-”Expected loss of social benefit (1) (*L*_1_)”-”Expected loss of social benefit (2) (*L*_2_)”)
(15) “fitness difference between supervision and no-supervision (*U_x_* − *U*_1−*x*_)” = “supervision fitness (*U_x_*)”—“no-supervision fitness (*U*_1−*x*_)”
(16) “changing rate of choosing supervision (*H* (*y*_1_, *y*_2_, *x*))” = “government supervision ratio (*x*)” * (1-”government supervision ratio (*x*)”) * “fitness difference between supervision and no-supervision (*U_x_* − *U*_1−*x*_)”

## Appendix C

The model equations of Figure 7.

Main equations of the contractor (1) making safety investment submodule:

(1) “contractor (1) making sufficient safety investment fitness (*U_y_*_1_)” = “Normal revenue of contractor (1) from regular safety production (*R*_1_)”—“Standard safety investment required in laws and regulations in contractor (1)’s program (*S*_1_)”
(2) “contractor (1) not making sufficient safety investment fitness (*U*_1-_*_y_*_1_)” = “probability of contractor (2) making sufficient safety investment (*y_2_*)” * (“Normal revenue of contractor (1) from regular safety production (*R*_1_)”—“Contractor (1)’s actual safety investment (*S*_1_’)”—“government supervision ratio (*x*)” * “Contractor (1)’s penalty (*P*_1_)”) + (1-”probability of contractor (2) making sufficient safety investment (*y_2_*)”) * (“Normal revenue of contractor (1) from regular safety production (*R*_1_)”—“Contractor (1)’s actual safety investment (*S*_1_’)”—“government supervision ratio (*x*)” * “Contractor’s penalty when both contractors do not making sufficient safety investment (*P*)”)
(3) “Contractor (1)’s penalty (*P*_1_)” = “penalty coefficient (*k*)” * (“Standard safety investment required in laws and regulations in contractor (1)’s program (*S*_1_)”—“Contractor (1)’s actual safety investment (*S*_1_’)”) * (1-”probability of contractor (1) making sufficient safety investment (*y*_1_)”) + “penalty coefficient (*k*)” * (“Standard safety investment required in laws and regulations in contractor (1)’s program (*S*_1_)”—“Contractor (1)’s actual safety investment (*S*_1_’)”)
(4) “Contractor’s penalty when both contractors do not make sufficient safety investment (*P*)” = “Contractor (2)’s penalty (*P*_2_)” + “Contractor (1)’s penalty (*P*_1_)
(5) “fitness difference between contractor (1) making sufficient safety investment and not making sufficient safety investment (*U_y_*_1_ − *U*_1__−*y*1_)” = “contractor (1) making sufficient safety investment fitness (*U_y_*_1_)”—“contractor (1) not making sufficient safety investment fitness (*U*_1__−*y*1_)”
(6) “changing rate of contractor (1) choosing making sufficient safety investment (*F* (*y*_1_, *y*_2_, *x*))” = “probability of contractor (1) making sufficient safety investment (*y*_1_)” * (1-”probability of contractor (1) making sufficient safety investment (*y*_1_)”) * “fitness difference between contractor (1) making sufficient safety investment and not making sufficient safety investment (*U_y_*_1_ – *U*_1–_*_y_*_1_)”

Main equations of the contractor (2) making safety investment submodule:

(7) “contractor (2) making sufficient safety investment fitness (*U_y_*_2_)” = “Normal revenue of contractor (2) from regular safety production (*R*_2_)”—“Standard safety investment required in laws and regulations in contractor (2)’s program (*S*_2_)”
(8) “contractor (2) not making sufficient safety investment fitness (*U*_1-_*_y_*_2_)” = “contractor (1) making sufficient safety investment ratio (*y*_1_)” * (“Normal revenue of contractor (2) from regular safety production (*R*_2_)”—“Cost of safety production to the contractor (2) not making sufficient safety investment (*S*_2_’)”—“government supervision ratio (*x*)” * “Contractor (2)’s penalty (*P*_2_)”) + (1-”contractor (1) making sufficient safety investment ratio (*y*_1_)”) * (“Normal revenue of contractor (2) from regular safety production (*R*_2_)”—“Contractor (2)’s actual safety investment (*S*_2_’)”—“government supervision ratio (*x*)” * “Contractor’s penalty when both contractors do not making sufficient safety investment (*P*)”)
(9) “Contractor (2)’s penalty (*P*_2_)” = “penalty coefficient (*k*)” * (“Standard safety investment required in laws and regulations in contractor (2)’s program (*S*_2_)”—“Contractor (2)’s actual safety investment (*S*_2_’)”) * (1-”probability of contractor (2) making sufficient safety investment (*y_2_*)”) + “penalty coefficient (*k*)” * (“Standard safety investment required in laws and regulations in contractor (2)’s program (*S*_2_)”—“Contractor (2)’s actual safety investment (*S*_2_’)”)
(10) “Contractor’s penalty when both contractors do not make sufficient safety investment (*P*)” = “Contractor (2)’s penalty (*P*_2_)” + “Contractor (1)’s penalty (*P*_1_)”
(11) “fitness difference between contractor (2) making sufficient safety investment and not making sufficient safety investment (*U_y_*_2_ – *U*_1–_*_y_*_2_)” = “contractor (2) making sufficient safety investment fitness (*U_y_*_2_)”—“contractor (2) not making sufficient safety investment fitness (*U*_1-_*_y_*_2_)”
(12) “changing rate of contractor (2) choosing making sufficient safety investment (*G* (*y*_1_, *y*_2_, *x*))” = “probability of contractor (2) making sufficient safety investment (y2)” * (1-”probability of contractor (2) making sufficient safety investment (*y_2_*)”) * “fitness difference between contractor (2) making sufficient safety investment and not making sufficient safety investment (*U_y_*_2_ – *U*_1–_*_y_*_2_)”

Main equations of the government supervision submodule:

(13) “supervision fitness (*U_x_*)” = “probability of contractor (1) making sufficient safety investment (*y*_1_)” * “probability of contractor (2) making sufficient safety investment (*y_2_*)” * (-“government safety supervision cost (*C_g_*)”) + “probability of contractor (1) making sufficient safety investment (*y*_1_)” * (1-“probability of contractor (2) making sufficient safety investment (*y_2_*)”) * (“Contractor (2)’s penalty (*P*_2_)”—“government safety supervision cost (*C_g_*)”—“Expected loss of social benefit (2) (*L*_2_)”) + (1-“probability of contractor (1) making sufficient safety investment (*y*_1_)”) * “probability of contractor (2) making sufficient safety investment (y2)” * (“Contractor (1)’s penalty (*P*_1_)”—“government safety supervision cost (*C_g_*)”—“Expected loss of social benefit (1) (*L*_1_)”) + (1-“probability of contractor (1) making sufficient safety investment (*y*_1_)”) * (1-“probability of contractor (2) making sufficient safety investment (*y_2_*)”) * (“Contractor’s penalty when both contractors do not make sufficient safety investment (*P*)”—“government safety supervision cost (*C_g_*)”—“Expected loss of social benefit (1) (*L*_1_)”—“Expected loss of social benefit (2) (*L*_2_)”)
(14) “no-supervision fitness (*U*_1-_*_x_*)” = “probability of contractor (1) making sufficient safety investment (*y*_1_)” * (1-”probability of contractor (2) making sufficient safety investment (*y_2_*)”) * (-”Expected loss of social benefit (2) (*L*_2_)”) + (1-”probability of contractor (1) making sufficient safety investment (*y*_1_)”) * “probability of contractor (2) making sufficient safety investment (*y_2_*)” * (-”Expected loss of social benefit(1) (*L*_1_)”) + (1-”probability of contractor (1) making sufficient safety investment (*y*_1_)”) * (1-”probability of contractor (2) making sufficient safety investment (*y_2_*)”) * (-”Expected loss of social benefit (1) (*L*_1_)”—“Expected loss of social benefit (2) (*L*_2_)”)
(15) “fitness difference between supervision and no-supervision (*U_x_* − *U*_1−_*_x_*)” = “supervision fitness (*U_x_*)”—“no-supervision fitness (*U*_1−_*_x_*)”
(16) “changing rate of choosing supervision (*H* (*y*_1_, *y*_2_, *x*))” = “government supervision ratio (*x*)” * (1-“government supervision ratio (*x*)”) * “fitness difference between supervision and no-supervision”

## Appendix D

**Table A1:** Nomenclature.

Classification	Nomenclature	Definition
Evolutionary game theory	ESS	Evolutionarily stable strategy, it is the basic concept of equilibrium in evolutionary games. The evolutionarily stable strategy is robust to small disturbances.
	Evolutionary game theory	Mathematical model studying strategic interaction between bounded rational decision-makers who do not have complete information.
	Fitness	In the theory of biological evolution, fitness is used to measure individual survival and reproductive opportunities, which is equal to payoff functions in economics.
	Player	Individuals or organizations that make independent decisions in a defined game.
	Replicator dynamics	A mechanism to describe the dynamic strategy adjustment process of bounded rational players.
	Strategy	Methods or practices chosen by game players when making a decision.
System dynamics	Auxiliary variable	Transition variables by calculation.
	External variable	Constants in the system.
	Level variable	Represent the system’s accumulations.
	Rate variable	Represent the flow in the system caused by the decision-making process.
	SD	System dynamics, a quantitative simulation method to analyze information feedback mechanism, which is often used to study complex systems
